# Two-year follow-up of gut microbiota alterations in patients after COVID-19: from the perspective of gut enterotype

**DOI:** 10.1128/spectrum.02774-24

**Published:** 2025-04-10

**Authors:** Qianhan Xie, Jiali Ni, Wanru Guo, Cheng Ding, Fengjiao Wang, Yechen Wu, Yuxi Zhao, Lingxiao Zhu, Kaijin Xu, Yanfei Chen

**Affiliations:** 1 State Key Laboratory for Diagnosis and Treatment of Infectious Diseases, National Clinical Research Center for Infectious Diseases, Collaborative Innovation Center for Diagnosis and Treatment of Infectious Diseases, The First Affiliated Hospital, College of Medicine, Zhejiang University 12377, Hangzhou, Zhejiang, China; 2 Jinan Microecological Biomedicine Shandong Laboratory 661980 , Jinan, Shandong, China; Children's National Hospital, George Washington University, Washington, DC, USA

**Keywords:** COVID-19, gut microbiota, Streptococcus, convalescence, pneumonia

## Abstract

Gut microbiota dysbiosis plays a role in the pathogenesis of post-acute coronavirus disease (COVID-19); however, the long-term recovery of the gut microbiota following SARS-CoV-2 infection remains insufficiently understood. In this study, 239 fecal samples were collected from 87 COVID-19 patients during the acute phase, and at 6 months, 1 year, and 2 years post-discharge. An additional 48 fecal samples from non-COVID-19 controls were also analyzed. Gut enterotypes were determined through 16S rRNA sequencing, and dynamic changes from the acute phase through recovery were assessed. Correlations between enterotypes and clinical characteristics were also examined. Two distinct enterotypes were identified: a Blautia-dominated enterotype (Enterotype-B) and a Streptococcus-dominated enterotype (Enterotype-S). Species diversity and richness were significantly higher in Enterotype-B. Enterotype-S, associated with inflammation, was more prevalent during the acute phase. Six months post-discharge, the ratio of Enterotype-B to Enterotype-S approached normal levels. Patients with Enterotype-S at admission had a higher incidence of severe cases during hospitalization and a longer duration of nasopharyngeal viral shedding compared with those with Enterotype-B. Furthermore, at 6 months post-discharge, residual pulmonary Computed Tomography (CT) abnormalities were more common in patients with Enterotype-S (55%) than in those with Enterotype-B (20%, *P* = 0.046). An index, B/S, representing the ratio of Blautia and Bifidobacterium to Streptococcus, was introduced and found to correlate closely with clinical characteristics. The Streptococcus-dominated enterotype is associated with inflammation and appears to influence both the severity of illness during the acute phase and cardiopulmonary recovery.

**IMPORTANCE:**

This study sheds new light on the intricate process of rehabilitating the gut microbiota following disruptions caused by COVID-19. Our approach, which examines the dynamics from the vantage point of enterotypes, reveals a more rapid recovery than previously reported, with the majority of the microbiota rebounding within a 6-month timeframe. Furthermore, our findings underscore the importance of the Blautia-dominated enterotype as a marker of gut health, which plays a pivotal role in mitigating the risk of severe progression and lingering effects post-SARS-CoV-2 infection. By scrutinizing these enterotypes, we can now foresee the potential severity and aftermath of COVID-19, offering a valuable tool for prognosis and intervention.

## INTRODUCTION

The severe acute respiratory syndrome coronavirus-2 (SARS-CoV-2) continues its global spread, with over 500 million infections and more than 6 million deaths reported worldwide since the onset of the COVID-19 pandemic (1). Although emerging strains have shown reduced virulence, the recovery process and the development of post-acute COVID-19 syndromes (PACS) have become growing concerns (2). A significant number of individuals recovering from COVID-19 experience long-term complications or persistent symptoms, as highlighted by mounting evidence (3, 4). However, the factors and mechanisms driving PACS remain poorly understood.

The human digestive tract is colonized by a multitude of microorganisms. In addition to the well-known *Helicobacter pylori*, there are many other types of microorganisms. These microorganisms are influenced by a variety of factors such as age, diet, and environment, and in turn, they also affect the health of the body (5). Recent studies have pointed to gut microbiota dysbiosis in COVID-19 patients, suggesting that microbial biomarkers could help differentiate patients by disease severity during hospitalization (6, 7). COVID-19 has been associated with an increased abundance of opportunistic pathogens such as *Clostridium hathewayi*, *Actinomyces viscosus*, *Bacteroides nordii*, and *Corynebacterium accolens*, alongside a depletion of beneficial commensals like *Faecalibacterium prausnitzii* and *Lachnospiraceae bacterium_5_1_63FAA* (6). Additionally, an elevated presence of *Clostridium ramosum* and *Clostridium hathewayi* and a reduction in *Faecalibacterium prausnitzii* and *Dorea longicatena* have been correlated with increased disease severity (6).

Notably, gut dysbiosis has been shown to persist beyond the clearance of SARS-CoV-2 and the resolution of respiratory symptoms, continuing even 6 months post-discharge—particularly in patients suffering from PACS (6, 8, 9). At this 6-month mark, the gut microbiomes of PACS patients were characterized by higher levels of *Ruminococcus gnavus* and *Bacteroides vulgatus*, with lower levels of *Faecalibacterium prausnitzii*. Patients with lingering respiratory symptoms (such as cough, sputum, nasal congestion, runny nose, or shortness of breath) exhibited a positive correlation with certain opportunistic pathogens, including *Streptococcus anginosus*, *Streptococcus vestibularis*, *Streptococcus gordonii*, and *Clostridium disporicum* (8). Given the close relationship between gut microbiota and pulmonary sequelae, as well as the limited treatment options for post-acute COVID-19 complications, understanding microbial changes during the recovery process could be critical for the early identification and treatment of PACS patients. An understanding of how gut dysbiosis contributes to PACS may lead to the development of probiotics or nutritional therapies that enhance recovery.

Gut dysbiosis during the acute phase of COVID-19 has been well documented in several studies; however, the recovery process of the gut microbiota following COVID-19 remains less understood. Many recovering patients continue to experience symptoms, a condition known as PACS or “Long COVID.” (10) Although the exact mechanisms driving PACS are still unclear, they may be linked to gut dysbiosis. Studies have shown that alterations in the gut microbiota can persist for up to 12 months after recovery from COVID-19 (11, 12). Mussabay et al. explored the fecal microbiota of patients in Kazakhstan during different post-COVID phases, identifying complex interactions between gut microbiota, their metabolites, and systemic cytokines that were associated with various post-COVID symptoms (13). The composition of the gut microbiota at the time of hospital admission was also found to correlate with the development of PACS (14). Beneficial bacteria like *Bifidobacterium pseudocatenulatum*, *Faecalibacterium prausnitzii*, *Roseburia inulinivorans*, and *Roseburia hominis* showed inverse correlations with PACS severity. The gut microbiome composition in PACS patients correlated with diverse symptoms, including respiratory, neuropsychiatric, gastrointestinal, dermatological, musculoskeletal, and fatigue. Opportunistic pathogens such as *Streptococcus anginosus*, *Streptococcus vestibularis*, *Streptococcus gordonii*, and *Clostridium disporicum* were linked to respiratory symptoms (14). These findings suggest that gut microbiota composition may play a key role in the persistence of symptoms and the development of PACS.

A major concern is that analyses based on gut microbiota diversity and structure suggest that the microbiota may not fully recover until 1 year post-recovery. However, the precise timeline for microbiota restoration remains unclear. Enterotype-based analysis offers a more comprehensive view of microbiota dynamics, assessing changes across the microbiome rather than focusing on individual bacterial species (15). In a cohort of post-COVID patients, with an average follow-up of 5 months post-infection, three distinct enterotypes were identified, each associated with different phenotypic outcomes (16). The microbiome of patients classified as Enterotype 1 (E1) was dominated by *Ruminococcus gnavus* and *Clostridium*, whereas Enterotype 2 (E2) was characterized by a predominance of *Faecalibacterium prausnitzii*, and Enterotype 3 (E3) was dominated by various species of *Bifidobacterium*. Patients in E1 reported a significantly higher incidence of respiratory symptoms, such as shortness of breath, cough, runny nose, and chest pain, compared with those in E2 and E3. Conversely, patients in E2 experienced a greater prevalence of neurological symptoms, including memory loss, difficulty concentrating, insomnia, and blurred vision, compared with the other enterotypes. In contrast, patients in E3 reported a significantly lower incidence of fatigue. However, this study was limited by its relatively short follow-up period and its reliance solely on self-reported symptoms, without any objective cardiopulmonary function assessments.

To investigate the long-term recovery of gut microbiota in patients following SARS-CoV-2 infection, we conducted a prospective cohort study of COVID-19 patients discharged in April 2020. These patients were systematically recruited and followed up for a period of 24 months. The primary objectives of this study were to (i) examine longitudinal changes in gut microbiota over the 2 years post-discharge, (ii) explore the associations between gut microbiota and pulmonary sequelae, and (iii) identify potential microbial biomarkers that could predict the development of residual lung abnormalities.

## MATERIALS AND METHODS

### Study design and participants

**Fig 1:**
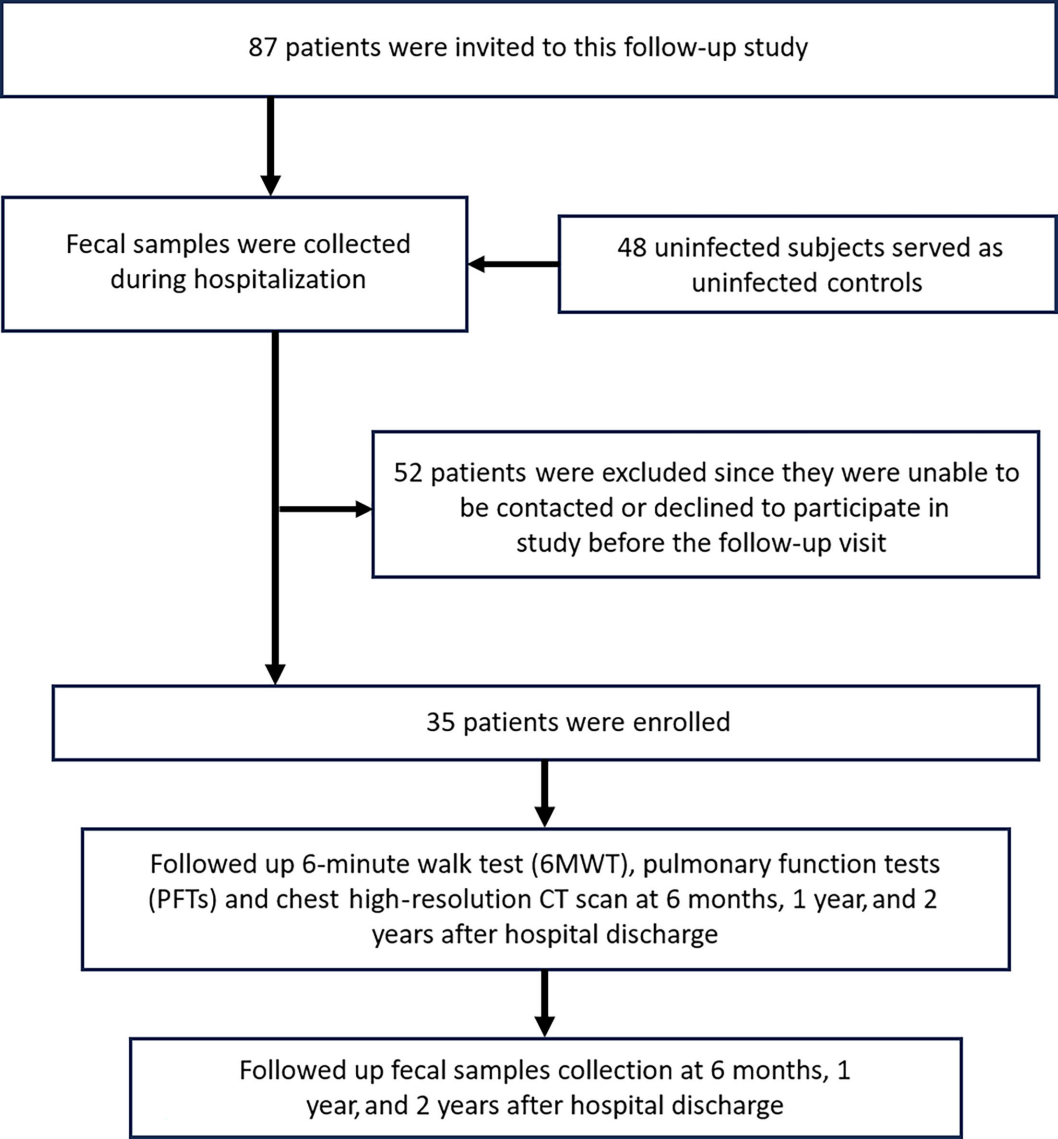
The schematic demonstrates the enrollment of participants and follow-up schedule.

This study was a prospective longitudinal follow-up of COVID-19 patients discharged from the First Affiliated Hospital, Zhejiang University School of Medicine, Hangzhou, China, between February 1 and March 15, 2020. These patients were followed up at 6 months, 1 year, and 2 years after discharge. During each follow-up visit, they underwent a 6-min walk test (6MWT), pulmonary function tests (PFTs), and a high-resolution chest CT scan. Additionally, fecal samples were collected at every follow-up appointment.(Fig. 1) Due to the limited understanding of PACS, we primarily referenced follow-up schedules from previous studies on SARS and other viral pneumonia patients during their recovery phase to set the time points for our research (17).

A control group of 48 uninfected individuals was used for comparison. The infected and control groups were matched for age, sex distribution, body mass index (BMI), and the presence of concomitant diseases. Exclusion criteria included a history of inflammatory bowel disease, gastrointestinal surgery, and the use of antibiotics, probiotics, or prebiotics within 1 month prior to the study. Since our primary focus is on the impact of COVID-19 on gut microbiota, these conditions were excluded from the study cohort for their potential influence on gut microbiota.

COVID-19 patients were classified according to the severity of their disease based on the Chinese clinical guidance for the diagnosis and treatment of COVID-19 pneumonia (7^th^ edition) (18). Patients were categorized into four levels of severity: mild, ordinary, severe, and critical. Mild illness was defined as having mild clinical symptoms without any radiologic signs of pneumonia. Ordinary illness included symptoms such as fever and respiratory issues, along with radiologic evidence of pneumonia. Severe illness was characterized by meeting at least one of the following criteria: a respiratory rate of ≥30 breaths per minute; arterial oxygen saturation (SaO2) ≤93% at rest; a PaO2/FiO2 ratio of ≤300 mmHg; or more than 50% progression of lesions on chest radiographs within 24–48 h. Critical illness cases were defined by any of the following: respiratory failure requiring mechanical ventilation, the occurrence of shock, or complications involving other organ failures necessitating intensive care. To streamline the analysis, mild and ordinary cases were combined into a “mild group,” whereas severe and critical cases were grouped together as a “severe group” for this study.

### DNA extraction and 16S rDNA sequencing

To mitigate the potential risk of infection from live viruses in stool samples and to ensure compliance with biosafety level 3 laboratory protocols, all experimental procedures were conducted in a biosafety level 2 laboratory. Previous studies have confirmed that for samples with low microbial abundance, there may be cross-contamination from reagents or buffers (19). However, since fecal samples typically have high microbial content, contamination during the extraction process is generally not a concern. No blank controls were included in the DNA extraction step. Fecal samples collected during the acute phase were heated at 56°C for 30 min to inactivate the virus. Samples obtained in the recovery phase and from control subjects were subjected to the same treatment to minimize methodological bias. Microbial DNA was extracted from the fecal samples using the PowerSoil Pro Kit (Qiagen, California, USA) in accordance with the manufacturer’s instructions. The bacterial 16S rRNA gene (V3–V4 region) was amplified using the primers 338F and 806R for sequencing. The PCR products were purified via electrophoretic separation on a 2.0% agarose gel using the AxyPrep DNA Gel Extraction Kit (Axygen Biosciences, Union City, CA, USA) and quantified using a Quantus Fluorometer (Promega, USA) following the manufacturer’s guidelines. The purified amplicons were then subjected to paired-end sequencing (2 × 300) using the Illumina MiSeq PE300 instrument (Illumina, San Diego, California, USA). Detailed procedures for bacterial genomic DNA extraction, PCR amplification, Illumina MiSeq sequencing, and bioinformatic analysis were conducted as previously described (7, 20). The raw sequence data reported in this paper have been deposited in the Genome Sequence Archive (Genomics, Proteomics, & Bioinformatics 2021) in National Genomics Data Center (Nucleic Acids Res 2022), China National Center for Bioinformation/Beijing Institute of Genomics, Chinese Academy of Sciences (GSA: CRA019806) that are publicly accessible at https://ngdc.cncb.ac.cn/gsa.

### Pulmonary function tests (PFTs)

Parameters including forced vital capacity (FVC), forced expiratory volume in the first second of expiration (FEV1), peak expiratory flow (PEF), FEV1/FVC ratio, diffusing capacity of the lung for carbon monoxide (DLCO), DLCO divided by alveolar volume (DLCO/VA), total lung capacity (TLC), residual volume (RV), and the ratio of residual volume to total lung capacity (RV/TLC) were measured using the SensorMedic Vmax System (USA). Data were reported as percentages of predicted normal values (21). Pulmonary function tests were not conducted for patients in the acute phase during hospitalization due to their weakness and the associated risk of cross-infection.

### Six-min walk distance test (6MWT)

At 6 months, 1 year, and 2 years after hospital discharge, the 6MWT was performed to estimate exercise capacity (22). The 6-min walk distance and vital signs before and after 6MWT were recorded. Heart rate, systolic and diastolic blood pressure, and pulse oximetry before the test were determined in the sitting position for at least 5 min under normal breathing.

### Radiological imaging

At 6 months, 1 year, and 2 years after hospital discharge, HRCT was performed to estimate residual radiological abnormality. Standardized techniques were applied to all radiographic examinations with the same CT equipment. Two radiologists with over 5 years of experience independently reviewed the CT images as described previously (23). Briefly, each lung was divided into three zones: superior (above the carina), middle (below the carina to the inferior pulmonary vein), and inferior (below the inferior pulmonary vein). Each lung zone (six lung zones in total) was scored according to the following rules: 0, 0% involvement; 1, less than 25% involvement; 2, 25%–50% involvement; 3, 50%–75% involvement; and 4, 75% involvement or higher. The sum of the total scores provided the overall lung involvement (the maximum value for both lungs was 24) (23).

### Statistical analyses

Continuous variables were compared between 6 months, 1 year, and 2-year follow-ups using pairwise Kruskal-Wallis test. If a statistically significant difference was obtained, the Nemenyi test is the method used for further pairwise multiple comparisons. The level of significance was set at 0.05 (two-sided). Statistical analyses were performed using SAS version 9.4 (SAS Institute Inc., Cary, NC, USA). Intergroup differences within taxa were determined using the Kruskal-Wallis H test followed by the Tukey-Kramer post-hoc test. False discovery rate correction (*P* < 0.05) was performed to reduce multiple comparison errors between groups. The clusterSim package in R was used to analyze enterotypes using the genus-level data as described previously (15). Briefly, the Jensen-Shannon divergence (JSD) between the samples was calculated, then the Partitioning Around Medoids algorithm was applied to cluster the samples, and the optimal number of clusters was determined based on the Calinski–Harabasz (CH) index, silhouette width, Davies-Bouldin (DBI) index, and Dunn index. The maximum index is selected as the optimal cluster number. The correlation between bacterial genera with an average relative abundance >1% and the results of PFTs and the 6MWT was evaluated using Spearman’s rank correlation coefficient. Absolute correlation coefficients higher than 0.4 were considered significant at *P* < 0.05. Sequence data were analyzed on the Majorbio Cloud Platform (www.majorbio.com).

## RESULTS

### Two gut enterotypes were identified among patients during the acute and recovery phases

**Fig 2:**
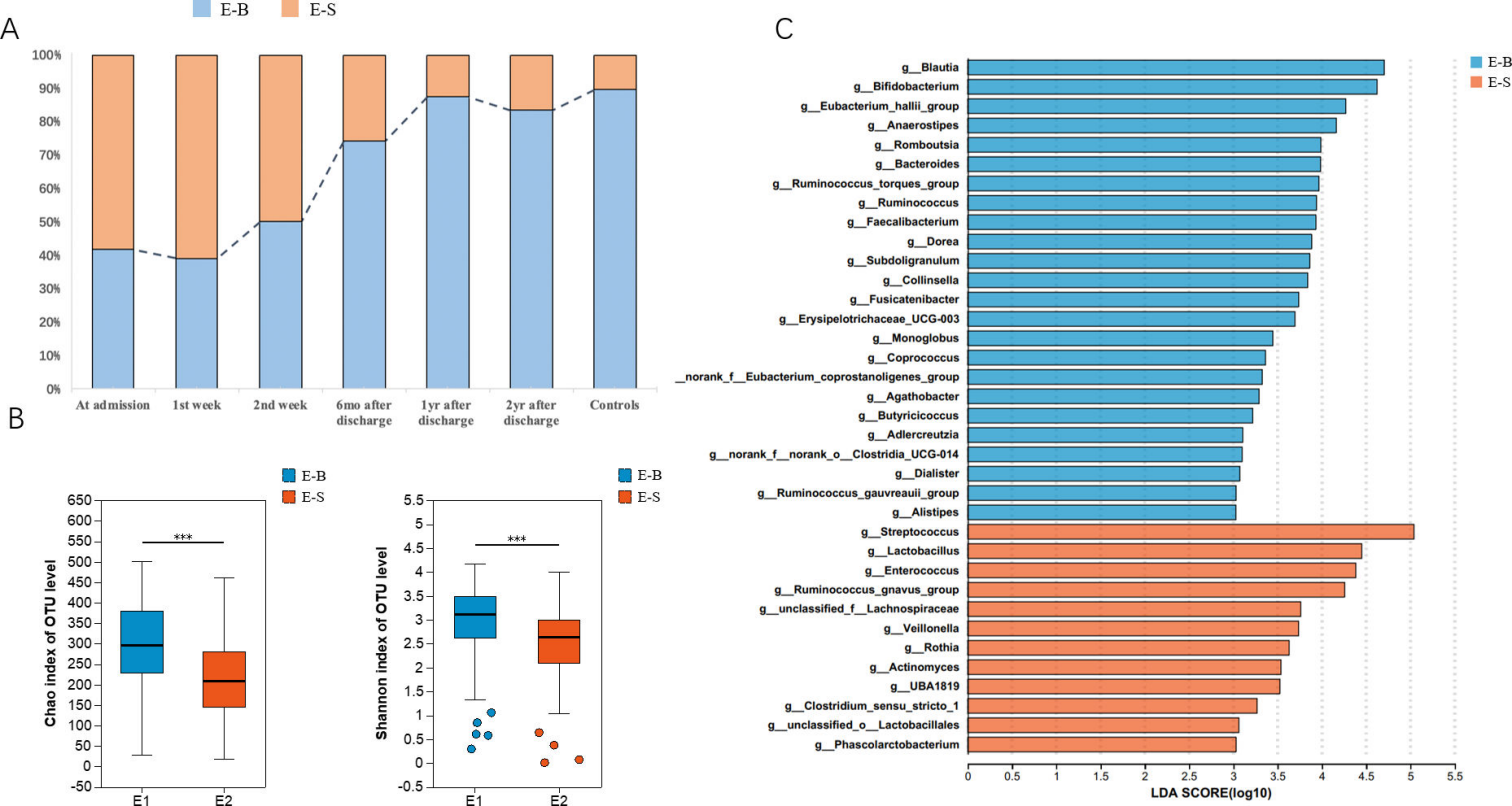
Two-year follow-up of gut enterotype alterations in patients after COVID-19. (A) Gut enterotype distribution of COVID-19 patients at different follow-up time points and healthy controls. (B) Comparison of microbial diversity and richness between Enterotype-B (E-B) and Enterotype-S (E-S). (C) Linear discrimination analysis (LDA) effect size (LEfSe) analysis was used to differentiate the Enterotype-B (E-B) and Enterotype-S (E-S) at the genus level. LDA scores showed significant differences in microbiota composition between Enterotype-B (blue) and Enterotype-S (orange). Only taxa with an LDA threshold >3 are shown.

Using the JSD distance metric, 287 samples were clustered according to the relative abundance of bacteria at the genus level. After evaluating the silhouette width, Calinski-Harabasz (CH) index, Davies-Bouldin index (DBI), and Dunn index, the optimal number of clusters was determined to be two (K = 2, see Fig. S1). These two enterotypes were classified according to the dominant bacteria present: Enterotype-S (*n* = 110), characterized by *Streptococcus*, and Enterotype-B (*n* = 177), dominated by *Blautia* and *Bifidobacterium*. Among the 48 uninfected controls, 41 individuals (85.4%) were categorized as Enterotype-B, whereas only 7 (14.6%) fell into Enterotype-S. Of the 239 samples collected from 87 patients during both the acute and recovery phases, 136 samples (56.9%) were identified as Enterotype-B, and 103 samples (43.1%) as Enterotype-S (Fig. 2A). The proportion of Enterotype-S in COVID-19 patients was significantly higher compared with that in the control group (*P* = 0.002).

The microbial diversity and richness were higher in Enterotype-B compared with Enterotype-S (Fig. 2B), as demonstrated by a significantly higher Chao1 index (Enterotype-B: 297 ± 95 vs. Enterotype-S: 214 ± 89, *P* < 0.001) and a significantly higher Shannon index in Enterotype-B (Enterotype-B: 2.98 ± 0.68 vs. Enterotype-S: 2.49 ± 0.74, *P* < 0.001). The dominant genera in each enterotype are depicted in Fig. 2C. In summary, *Streptococcus*, *Lactobacillus*, and *Enterococcus* were more abundant in Enterotype-S, whereas *Blautia*, *Bifidobacterium*, and the *Eubacterium hallii* groups were more prevalent in Enterotype-B.

Further analysis of microbial diversity and richness between the two enterotypes revealed notable differences at the phylum level: Enterotype-B had higher levels of Actinobacteriota and Bacteroidota, but lower levels of Firmicutes, Proteobacteria, and Fusobacteria compared to Enterotype-S. At the genus level, Enterotype-B was enriched with potentially beneficial bacteria such as *Blautia*, *Bifidobacterium*, the *Eubacterium hallii group*, *Anaerostipes*, *Romboutsia*, *Bacteroides*, *Ruminococcus*, *Faecalibacterium*, and others. In contrast, Enterotype-S showed a higher abundance of potentially pathogenic bacteria, including *Streptococcus*, d, and others (Fig. 2C).

### Longitudinal alterations of enterotype from acute phase to convalescence

To further investigate the dynamic recovery process of enterotypes, we compared the distribution of the two enterotypes in COVID-19 patients at different stages of illness. Of the 136 Enterotype-B samples, 73 (53.7%) were from the acute phase and 63 (46.3%) from the recovery phase. Among the 103 Enterotype-S samples, 89 (86.4%) were from the acute phase, whereas only 14 (13.6%) were from the recovery phase. Enterotype-S appeared to be associated with inflammation and was more representative of the gut microbiota during the acute phase. The proportion of Enterotype-S was significantly higher in the acute phase compared with uninfected controls. At 6 months (Enterotype-B *n* = 23, Enterotype-S *n* = 8), 12 months (Enterotype-B *n* = 21, Enterotype-S *n* = 3), and 2 years (Enterotype-B *n* = 10, Enterotype-S *n* = 2) post-discharge, the enterotype distribution was similar to that of uninfected controls. The proportions of Enterotype-B and Enterotype-S at 6 months after hospital discharge were comparable with those in uninfected individuals, suggesting that the gut microbiota had largely returned to a normal state during the recovery period (Fig. 2A).

### The enterotype of the acute phase affects illness severity during the acute phase

We compared the clinical characteristics of patients with different enterotype statuses at admission (Table 1). Patients in the acute phase with Enterotype-B (median age: 48, interquartile range [IQR]: 41–57) were significantly younger (*P* < 0.05) than those with Enterotype-S (median age: 55, IQR: 44–64). Additionally, the duration of nasopharyngeal viral RNA shedding was significantly shorter in patients with Enterotype-B (median: 15 days, IQR: 12–18) compared with those with Enterotype-S (median: 19 days, IQR: 14–26) (*P* = 0.01). Patients were classified as having mild or severe illness based on the severity of their condition during hospitalization. There were 17 severe cases (51.5%) among patients with Enterotype-B at admission, a significantly lower proportion than that seen in patients with Enterotype-S, where 39 individuals (84.8%) had severe illness (*P* < 0.05). Although the incidence of secondary bacterial infections was slightly higher in Enterotype-S patients, the difference was not statistically significant (*P* = 0.09). No significant differences were observed between the two enterotypes with respect to gender, body weight, smoking history, alcohol consumption, underlying conditions, use of assisted ventilation, presence of gastrointestinal symptoms, or bacterial infections (*P* > 0.05).

**TABLE 1:** Comparison of acute phase clinical characteristics of patients with different enterotypes at admission^*a*^

	Enterotype-B patients (*n* = 33)	Enterotype-S patients (*n* = 46)	*P* value
Age, years	48 (41, 57)	55 (44.25, 64)	0.040*^b^
Male, n (%)	18 (54.5%)	30 (65.2%)	0.338
BMI, kg/m^2^	24.3 (22, 26.7)	24.2 (21.2, 25.5)	0.527
Smoking history	5 (15.2%)	3 (6.5%)	0.381
Drinking history	3 (9.1%)	3 (6.5%)	1.000
Comorbidities			
Hypertension, n (%)	7 (21.2%)	15 (32.6%)	0.265
Type 2 diabetes, n (%)	2 (6.1%)	7 (15.2%)	0.366
Coronary artery heart disease, n (%)	0 (0.0%)	3 (6.5%)	0.369
Illness progress			
Digestive tract symptom	2 (6.1%)	7 (15.2%)	0.366
Positive fecal viral RNA	14 (42.4%)	20 (43.5%)	0.926
Nasopharyngeal viral RNA shedding duration, d	15 (12, 18)	19 (14.3, 26)	0.007*
Secondary bacterial infection	1 (3.0%)	8 (18.2%)	0.091
Severe illness during hospitalization	17 (51.5%)	39 (84.8%)	0.001*
Mechanical ventilation, n (%)	1 (3.0%)	1 (2.3%)	1.000
HFNC, n (%)	2 (6.1%)	7 (15.9%)	0.331

^*a*^ The quantitative data are shown as median data and inter quartilerange data in brackets. The occurrence data are shown as no. (%). Values indicate no. of positive results/total no. of patients with available assay results. Student’s t-test or Mann-Whitney U test were used for the quantitative data when applicable. Fisher's exact test was used for category data with *P* < 0.05 as significant. *P <0.05. Abbreviations: BMI, body mass index; HFNC, high flow nasal catheter oxygen therapy.

^*b*^ Statistically significance with a *P*-value ≤0.05 was marked as bold.

### Enterotype of the acute phase affects illness recovery

Post-COVID-19 sequelae were assessed through pulmonary high-resolution CT (HRCT), pulmonary function tests (PFTs), and 6-min walk distance tests at 6 months (*n* = 39), 1 year (*n* = 34), and 2 years (*n* = 16) after discharge. The recovery characteristics were compared based on the initial enterotype at admission. At 6 months post-discharge, the proportion of patients with residual pulmonary abnormalities on CT was significantly lower in Enterotype-B (20%) compared with Enterotype-S (55%) (*P* = 0.046) (Table S1). However, no significant differences in HRCT findings were observed between the two groups during the other follow-up visits. At 6 months post-discharge, patients with Enterotype-B had a significantly lower pre-walk heart rate (median: 74, IQR: 71–78) compared with those with Enterotype-S (median: 85, IQR: 83–88) (*P* < 0.05) (Table S1). One year after discharge, patients with Enterotype-B showed significantly higher peak expiratory flow (PEF) (median: 96, IQR: 82–101 vs. median: 73, IQR: 65–83, *P* < 0.05) and maximal expiratory flow at 25% of vital capacity (MEF25%) (median: 102, IQR: 79–108 vs. median: 82, IQR: 55–96, *P* < 0.05) compared with Enterotype-S (Table 2). Additionally, systolic blood pressure before and after the 6-min walk test was significantly lower in Enterotype-B (before: median: 123, IQR: 114–134; after: median: 128, IQR: 121–136) than in Enterotype-S (before: median: 140, IQR: 132–154; after: median: 138, IQR: 130–155) (*P* < 0.05). Two years after discharge, no significant differences between the two enterotypes were observed (Table S2).

**TABLE 2:** Comparison of the recovery phase (1 year) clinical characteristics of patients with different enterotypes at admission^*a*^

	Enterotype-B patients (*n* = 17)	Enterotype-S patients (*n* = 22)	*P* value
PFTs			
FVC	99 (95, 102)	86 (79,105)	0.140
FEV1	100 (98, 104)	88 (76, 102)	0.085
PEF	96 (82,101)	73 (65, 83)	**0.041**
FEV1/FVC%	84.6 (78.3, 86.3)	80.4 (74.1, 83.6)	0.099
DLCO	92 (69, 108)	69 (53, 88)	0.099
TL C(DLCO)	89 (84, 95)	78 (69, 95)	0.150
FEF25-75%	112 (103,124)	112 (95,120)	0.658
MEF 75%%	97 (93, 108)	96 (86, 104)	0.205
MEF 50%%	102 (83,118)	89 (61,109)	0.160
MEF 25%%	102 (79,108)	82 (55, 96)	**0.011**
MVV	87 (76, 113)	77 (55, 96)	0.170
DLCO/VA	85 (67, 96)	71 (65,103)	0.433
IV C(DLCO)	100 (96, 107)	103 (96, 108)	0.760
RV(DLCO)	121 (109,140)	120 (105, 127)	0.454
RV/TLC(DLCO)	124 (107, 127)	126 (119,137)	0.394
DLCO meann	112 (103,124)	112 (95,120)	0.658
Exercise capacity			
Pre-walk heart rate	80 (73, 90)	82.5 (75, 89)	0.769
Pre-walk systolic blood pressure	123 (114, 134)	140 (132, 154)	**0.015**
Pre-walk diastolic bloodpressuree	84 (77, 87)	87 (82, 90)	0.262
Pre-walk O2 saturation, %	99 (99,100)	99 (97, 100)	0.399
Six-min walk distances, m	620 (570,640)	570 (560,610)	0.377
Post-walk O_2_ saturation, %	98 (98, 99)	98 (97, 98)	0.200
Post-walk systolic blood pressure	128 (121,136)	138 (130, 155)	**0.012**
Post-walk diastolic blood pressure	83 (76, 86)	86.5 (81, 90)	0.092
Post-walk heart rate	105 (99,112)	104.5 (95,115)	0.953
*HRCT*	*n* = 17	*n* = 14	
Severe imaging findings of HRCT	2(11.8%)	5(35.7%)	0.198

^*a*^ The quantitative data are shown as median data and IQR data in brackets. The occurrence data are shown as no. (%). Values indicate no. of positive results/total no. of patients with available assay results. Between- group comparisons of continuous variables were tested by Mann-Whitney U test or Fisher’s exact test. Statistically significance with a *P*-value ≤0.05 was marked as bold. A *P*-value ≤0.05 was denoted as statistically significant. Pulmonary function tests were expressed as a percent of the predicted value. MEF 25%, mean expiratory flow at 25%; MEF 50%, mean expiratory flow at 50%; MEF 75%, mean expiratory flow at 75%; DLCO, diffusing capacity of the lung for carbon monoxide; DLCO/VA, diffusing capacity divided by the alveolar volume; FEF25%–75%, forced expiratory flow at 25%–75%; FEV1, forced expiratory volume in the first 1 s of expiration; FVC, forced vital capacity; IVC, inspiratory vital capacity; MVV, maximal voluntary ventilation; PEF, peak expiratory flow; PFTs, pulmonary function tests; RV, residual volume; RV/TLC, residual volume divided by the total lung capacity; TLC, total lung capacity.

### B/S value is correlated with COVID-19 severity and recovery

LEfSe analysis identified *Blautia*, *Bifidobacterium*, and *Streptococcus* as the bacterial genera with the most significant differences between Enterotype-B and Enterotype-S (LDA score >4). Based on these findings, a B/S index was calculated by taking the ratio of the relative abundance of *Blautia* and *Bifidobacterium* to *Streptococcus*, and this index was correlated with clinical characteristics. During the acute phase, patients with mild illness had significantly higher B/S values than those with severe illness (median: 12.17, IQR: 1.27–82.41 vs. median: 1.26, IQR: 0.30–3.12, *P* = 0.001). Spearman correlation analysis showed a weak negative correlation between the B/S value and viral shedding duration during the acute phase (r = −0.251, *P* = 0.026). Additionally, residual pulmonary CT abnormalities at 6 months post-discharge were negatively correlated with the B/S value (r = −0.341, *P* = 0.045). At 1 year post-discharge, significant positive correlations were observed between the B/S value and pulmonary function indices, such as peak expiratory flow (PEF) (r = 0.315, *P* = 0.050) and maximal expiratory flow at 75% of vital capacity (MEF75%) (r = 0.378, *P* = 0.018). The post-walk heart rate was negatively correlated with the B/S value at 1 year after discharge (Table 3).

**TABLE 3:** Analysis of correlation between B/S value and clinical characteristics of different phases of patients^*a*^

	B/S	
	*P* value	r (Spearman's rho)
PFTs (1 year)		
FVC	0.651	0.075
FEV1	0.331	0.160
PEF	0.050	0.315
FEV1/FVC%	0.161	0.229
DLCO	0.707	0.062
TL C(DLCO)	0.446	0.126
FEF 25%–75%	0.284	0.176
MEF 75%	0.018	0.378
MEF 50%	0.378	0.145
MEF 25%	0.323	0.163
MVV	0.742	0.054
DLCO/VA	0.455	−0.123
IVC (DLCO)	0.211	0.205
RV (DLCO)	0.642	0.077
RV/TLC (DLCO)	0.204	−0.208
DLCO mean	0.707	0.062
Exercise capacity (1 year)		
Pre-walk heart rate	0.154	−0.262
Pre-walk systolic blood pressure	0.069	−0.331
Pre-walk diastolic bloodpressuree	0.453	−0.140
Pre-walk O2 saturation, %	0.146	0.268
Six-min walk distances, m	0.691	−0.074
Post-walk O_2_ saturation, %	0.161	0.258
Post-walk systolic blood pressure	0.106	−0.196
Post-walk diastolic blood pressure	0.241	−0.217
Post-walk heart rate	0.049	−0.357
6 months after hospital discharge		
The residual pulmonary CT abnormalities	0.045	−0.341
Acute phase		
Viral shedding duration at the acute phase	0.026	−0.251

^*a*^ The occurrence data are shown as no. (%). Values indicate no. of positive results/total no. of patients with available assay results. Spearman correlation analysis was used to explore whether there was a relationship between the B/S value and the data of subjects at different periods. Statistically significant results with a *P*-value ≤0.05 were marked as bold. A *P*-value ≤0.05 was denoted as statistically significant. r < 0 indicates negative correlation, r = 0 indicates no correlation, and r > 0 indicates positive correlation. Pulmonary function tests were expressed as a percent of the predicted value. MEF 25%, mean expiratory flow at 25%; MEF 50%, mean expiratory flow at 50%; MEF 75%, mean expiratory flow at 75%; DLCO, diffusing capacity of the lung for carbon monoxide; DLCO/VA, diffusing capacity divided by the alveolar volume; FEF 25%–75%, forced expiratory flow at 25%–75%; FEV1, forced expiratory volume in the first 1 s of expiration; FVC, forced vital capacity; IVC, inspiratory vital capacity; MVV, maximal voluntary ventilation; PEF, peak expiratory flow; PFTs, pulmonary function tests; RV, residual volume; RV/TLC, residual volume divided by the total lung capacity; TLC, total lung capacity; 6WMT, 6 min walk tests.

## DISCUSSION

Gut dysbiosis during acute COVID-19 infection has been well documented in several studies, but little is known about the recovery process of the gut microbiota post-COVID-19 or its association with post-acute pulmonary sequelae. This study aimed to provide a longitudinal characterization of the gut microbiota for up to 2 years after hospital discharge. Our findings suggest that the gut microbial enterotype generally returns to normal within 6 months post-SARS-CoV-2 infection. Furthermore, the gut enterotype during the acute phase of SARS-CoV-2 infection can influence both the severity of the illness and the recovery process. The B/S index, which reflects the ratio of the relative abundance of *Blautia* and *Bifidobacterium* to *Streptococcus*, may serve as a simple predictive marker for disease prognosis.

Previous studies have shown that COVID-19 infection alters the composition and diversity of gut microbiota, with significant changes observed during the acute phase of the illness (6, 7). These alterations may persist even after viral clearance (8, 11). However, these findings are primarily based on the diversity and richness of microbial genera and species within the community. Given the substantial interindividual variation in gut microbiota, focusing too heavily on specific bacterial genera or species may risk misinterpreting the overall microbial landscape. Enterotype analysis, which classifies individuals into distinct microbial community types based on their dominant gut bacteria, offers a clearer understanding of interindividual differences in gut microbiota (15). Our research is the first to examine the impact of COVID-19 on gut microbiota from the perspective of enterotypes. We identified two enterotypes: one dominated by *Blautia* and the other by *Streptococcus*. These findings align with previous studies that identified three enterotypes—*Bacteroides*, *Blautia*, and *Streptococcus*-dominated clusters in the human gut (24). The underrepresentation of the *Bacteroides*-dominated enterotype in our study could be due to the heat treatment of fecal samples (56°C for 30 min) to inactivate the virus, which may have affected the bacterial composition. Using microbial enterotype-like cluster analysis, our results indicated that the distribution of gut enterotypes generally returned to normal at 6 months post-infection. These findings suggest that the impact of SARS-CoV-2 on gut microbiota may be less pronounced than initially thought, with the gut microbiota showing a tendency to return to homeostatic equilibrium relatively quickly (12, 25).

It has been widely accepted that robust gut microbiota plays a pivotal role in combating viral infections (26). Our findings also demonstrated that patients with a healthier enterotype (Enterotype-B) tend to experience milder symptoms and recover more quickly from COVID-19. Previous studies have suggested that a diverse and balanced gut microbiome enhances the body’s immune response against various viruses, including influenza, norovirus, and respiratory syncytial virus (27, 28). The microbial alpha diversity of Enterotype-B is significantly higher than that of Enterotype-S. Alpha diversity is an important indicator of the health of microbial community structure, with higher alpha diversity reflecting a more robust and stable microbiota composition (29). Enterotype-B was predominantly enriched with *Blautia*, *Bifidobacterium*, *Eubacterium hallii* group, *Anaerostipes*, *Romboutsia*, *Bacteroides*, *Ruminococcus*, *Faecalibacterium,* and other potentially beneficial bacteria. Species of *Bifidobacterium* have been shown to directly inhibit viral replication and boost antiviral immunity (30). Additionally, many of the bacteria overrepresented in Enterotype-B produce metabolites such as short-chain fatty acids (SCFAs), which are critical in regulating immune cell function and enhancing antiviral defenses (31). These findings highlight the importance of maintaining a healthy gut microbiome to support immune defenses against viral pathogens. Factors related to host genetics, ethnicity, lifestyle, diet, and geographic location collectively shape a healthy gut microbiota, thereby influencing an individual’s susceptibility to infectious diseases (32). A recent study showed that microbial responses to SARS-CoV-2 infection vary widely, with severe cases being associated with *Enterococcus faecium* and *Akkermansia muciniphila*, whereas milder cases were linked to *Faecalibacterium prausnitzii*, *Alistipes putredinis*, *Blautia faecis*, and other species (33). Our results are consistent with these findings, but we believe that differences in the gut microbiota response between mild and severe cases are not solely determined by illness severity. Instead, the baseline composition of the gut microbiota plays a significant role in shaping the disease trajectory.

Several previous studies have reported an association between the gut microbiome and the occurrence and phenotypic manifestations of PACS (14, 16). Specifically, patients with PACS tend to have higher levels of *Ruminococcus gnavus*, *Bacteroides vulgatus*, and lower levels of *Faecalibacterium prausnitzii* (14). Consistent with these findings, our study suggests that the gut enterotype during the acute phase is linked to the recovery rate of pulmonary function and the presence of radiological abnormalities. Six months after discharge, the percentage of patients with residual pulmonary CT abnormalities was significantly lower in those with Enterotype-B (20%) compared with those with Enterotype-S (55%) (*P* = 0.046). Our study is among the first to correlate radiological abnormalities and pulmonary function in COVID-19 patients with their gut microbiota. These results add to previous findings, which were mostly based on subjective symptom assessments through surveys. By comparing the cardiopulmonary function and lung imaging of patients with different gut enterotypes during the recovery phase, our study provides objective evidence for the influence of gut microbiota on COVID-19 recovery, offering a novel perspective on the role of the gut microbiome in post-COVID outcomes.

We developed an index B/S value, calculated as the ratio of the relative abundance of *Blautia* and *Bifidobacterium* to *Streptococcus*, which demonstrated a strong correlation with COVID-19 severity during both the acute and recovery phases. The B/S value was negatively correlated with viral shedding duration during the acute phase and residual pulmonary CT abnormalities during recovery. *Bifidobacterium*, a well-known probiotic, has shown promise in improving symptoms during the acute phase of COVID-19 in several studies (34–36). *Blautia*, a strong candidate for next-generation probiotics, has potential benefits in regulating host metabolism, as a traditional probiotic strain *Lactobacillus* species (37, 38). Additionally, *Blautia* has been shown to stimulate intestinal mucus secretion, supporting the integrity of the intestinal mucosal barrier (39). Several studies have reported a decrease in the abundance of *Bifidobacterium* and *Blautia* during SARS-CoV-2 infection (14, 40, 41). Co-occurrence network analysis further confirmed that *Blautia* is negatively correlated with pro-inflammatory bacteria such as *Ruminococcus*, suggesting its potential role in mitigating inflammation (42). *Blautia* could be considered a future probiotic treatment for pneumonia patients (43). Our study not only confirmed the predictive value of Blautia for COVID-19 severity but also, for the first time, established a link between Blautia and the recovery of cardiopulmonary function post-COVID-19. This provides new tools and methods for monitoring and treating post-COVID-19 sequelae, particularly in relation to lung health and overall recovery.

Our study has several limitations. First, the sample size was relatively small, and the single-center design may limit the generalizability of our findings. Second, although inflammatory cytokines are known to play a critical role in the pathogenesis of COVID-19 and are associated with disease severity and prognosis, our study did not include measurements of inflammatory factors. Incorporating cytokine profiling data could provide valuable insights into the relationship between gut microbiota alterations and the inflammatory status or disease progression in COVID-19 patients (44, 45). Third, the dynamic oral-gastric microbial axis, which connects oral and gastrointestinal health, has not been fully explored in our study (46, 47). Given that the oral cavity serves as an entry point for SARS-CoV-2 and is closely linked to the respiratory system, further investigation into the role of oral microbiota in COVID-19 is warranted to better understand the multisystem impact of the disease. Finally, based on the current findings, we can only conclude that individuals with Enterotype B exhibit faster recovery rates. Although this correlation suggests that gut microbiota could serve as a potential target for early diagnosis, the causal relationship requires further validation in future studies.

### Conclusion

We identified two enterotypes, the Blautia-dominated enterotype and the Streptococcus-dominated enterotype in patients after COVID-19. The Streptococcus-dominated enterotype appeared to be an inflammatory-associated enterotype more representative of the gut microbiota during the acute phase of COVID-19. The Streptococcus-dominated enterotype of the acute phase could affect illness severity during the acute phase, as well as cardiopulmonary recovery after COVID-19. Future research should focus on exploring targeted interventions, such as probiotics or microbiota modulation therapies to reduce inflammation and improve clinical outcomes in patients after COVID-19.

## Data Availability

The raw sequence data reported in this paper have been deposited in the Genome Sequence Archive (Genomics, Proteomics & Bioinformatics 2021) in National Genomics Data Center (Nucleic Acids Res 2022), China National Center for Bioinformation / Beijing Institute of Genomics, Chinese Academy of Sciences (GSA: CRA019806) that are publicly accessible at https://ngdc.cncb.ac.cn/gsa.

## SUPPLEMENTAL MATERIAL

Supplemental figure and tables spectrum.02774-24-s0001.docx Figure S1; Tables S1 and S2.

ASM does not own the copyrights to Supplemental Material that may be linked to, or accessed through, an article. The authors have granted ASM a non-exclusive, world-wide license to publish the Supplemental Material files. Please contact the corresponding author directly for reuse.
