# Supplementary material for: Two-year follow-up of gut microbiota alterations in patients after COVID-19: from the perspective of gut enterotype
Source: Microbiol Spectr. 2025 Apr 10;13(5):e02774-24. doi: 10.1128/spectrum.02774-24 (PMC12054050; doi:10.1128/spectrum.02774-24)
Supplement: Supplemental figure and tables — Figure S1; Tables S1 and S2. [file spectrum.02774-24-s0001.docx]

**Supplementary material**

**Supplementary Figure S1.** Two enterotypes clusters were identified with the highest Calinski–Harabasz.

**
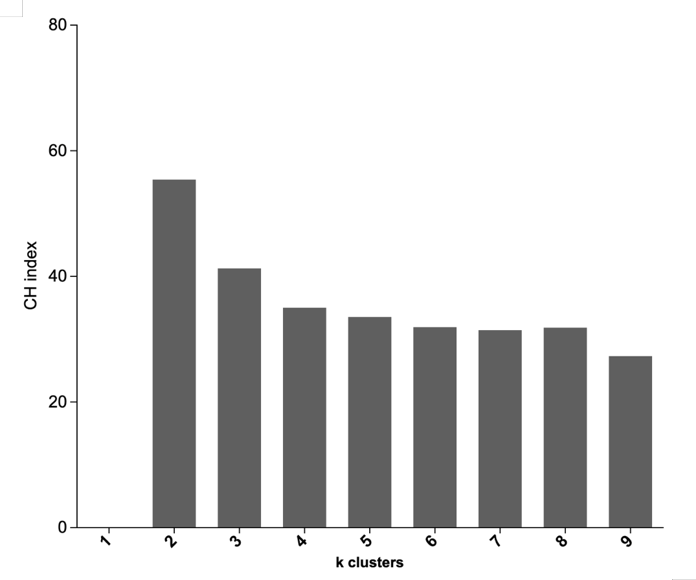
**

**Supplementary Table S1.** Comparative of clinical characteristics during recovery phase (6 Months Post-COVID-19) in patients with different enterotypes at admission

|  | Enterotype-B patients(n=17) | Enterotype-S patients(n=22) | P-value |
| --- | --- | --- | --- |
| *Pulmonary function tests* |  |  |  |
| FVC | 103 (98,107) | 94 (85,114) | 0.590 |
| FEV1 | 102 (93,107) | 95.5 (79,113) | 0.524 |
| PEF | 84 (79,100) | 81 (71,101) | 0.470 |
| FEV1/FVC% | 80.7 (74,87.3) | 81.35 (77,87.9) | 0.734 |
| DLCO | 95 (78,99) | 86 (76,94) | 0.169 |
| TLC(DLCO) | 100 (95,106) | 96 (86,106) | 0.275 |
| FEF25-75% | 91 (65,118) | 89 (65,130) | 0.713 |
| MEF75% | 90 (80,110) | 86 (71,111) | 0.469 |
| MEF50% | 89 (73,106) | 80.5 (79,126) | 1.000 |
| MEF25% | 76 (59,105) | 77.5 (57,137) | 0.620 |
| MVV | 88 (75,103) | 87 (63,107) | 0.755 |
| DLCO/VA | 74 (69,79) | 73 (66,87) | 0.820 |
| IVC(DLCO) | 88 (79,92) | 82.5 (68,95) | 0.364 |
| RV(DLCO) | 120 (106,132) | 119.5 (106,130) | 0.660 |
| RV/TLC(DLCO) | 124 (112,138) | 125.5 (107,151) | 0.702 |
| DLCOmean | 95 (77,99) | 85 (74,93) | 0.137 |
| *Exercise capacity* |  |  |  |
| Pre-walk heart rate | 74 (71,78) | 85 (83,88) | **0.025** |
| Pre-walk systolic blood pressure | 120 (114,136) | 133 (121,146) | 0.281 |
| Pre-walk diastolic blood presure | 71 (69,89) | 81 (71,87) | 0.680 |
| Pre-walk O2 saturation, % | 98 (98,99) | 98 (97,99) | 0.680 |
| Six-min walk distances, m | 610 (580,652) | 560 (520,640) | 0.145 |
| Post-walk O_2_ saturation, % | 98 (97,98) | 98 (97,99) | 0.869 |
| Post-walk systolic blood pressure | 126 (114,136) | 131 (122,148) | 0.086 |
| Post-walk diastolic blood presure | 77 (75,84) | 80 (69,89) | 0.805 |
| Post-walk heart rate | 112 (99,114) | 102 (98,112) | 0.563 |
| *HRCT* |  |  |  |
| Severe imaging findings of HRCT | 3(20%) | 11(55%) | **0.046** |

The quantitative data are shown as median data and IQR data in brackets.

The occurrence data are shown as no. (%). Values indicate no. of positive results/total no. of patients with available assay results.

Between- group comparisons of continuous variables in patients with low and high richness were tested by Kruskal- Wallis test.

Statistically significance with a p-value ≤0.05 was marked as bold.

A p-value ≤0.05 was denoted as statistically significant.

Pulmonary function tests were expressed as a percent of the predicted value.

MEF 25%, mean expiratory flow at 25%; MEF 50%, mean expiratory flow at 50%; MEF 75%, mean expiratory flow at 75%; DLCO, diffusing capacity of the lung for carbon monoxide; DLCO/VA, diffusing capacity divided by the alveolar volume; FEF25%–75%, forced expiratory flow at 25%–75%; FEV1, forced expiratory volume in the first 1 s of expiration; FVC, forced vital capacity; IVC, inspiratory vital capacity; MVV, maximal voluntary ventilation; PEF, peak expiratory flow; PFTs, pulmonary function tests; RV, residual volume; RV/TLC, residual volume divided by the total lung capacity; TLC, total lung capacity.

**Supplementary Table S2.** Comparative of clinical characteristics during recovery phase (2 years Post-COVID-19) in patients with different enterotypes at admission

|  | Enterotype-B patients(n=9) | Enterotype-S patients(n=7) | P-value |
| --- | --- | --- | --- |
| *Pulmonary function tests* |  |  |  |
| FVC | 95 (93,101) | 86 (83.5,101) | 0.252 |
| FEV1 | 99 (91,101) | 80 (80,96.5) | 0.174 |
| PEF | 90 (86,99) | 91 (79.5,98) | 0.681 |
| FEV1/FVC% | 104 (97,109) | 99 (97,102.5) | 0.210 |
| DLCO | 95 (89,103) | 94 (90,102.5) | 0.681 |
| TLC(DLCO) | 97 (94,105) | 88 (83.5,99) | 0.091 |
| FEF25-75% | 82 (70,102) | 63 (59,85) | 0.174 |
| MEF75% | 97 (89,106) | 93 (71,102.5) | 0.470 |
| MEF50% | 84 (72,103) | 71 (57,87,5) | 0.174 |
| MEF25% | 75 (54,95) | 51 (46.5,66.5) | 0.174 |
| MVV | 97 (93,102) | 88 (79.5,104) | 0.351 |
| DLCO/VA | 99 (93,107) | 114 (99,117.5) | 0.408 |
| IVC(DLCO) | 92 (88,101) | 80 (76,96) | 0.174 |
| RV(DLCO) | 110 (99,124) | 104 (98,108) | 0.174 |
| RV/TLC(DLCO) | 112 (109,117) | 114 (107.5,115) | 0.681 |
| DLCOmean | 95 (89,103) | 94 (90,102.5) | 0.681 |
| *Exercise capacity* |  |  |  |
| Pre-walk heart rate | 76 (72,77) | 77 (76,84) | 0.252 |
| Pre-walk systolic blood pressure | 118 (114,135) | 140 (125,146.5) | 0.055 |
| Pre-walk diastolic blood presure | 77 (71,78) | 88 (82,90) | 0.174 |
| Pre-walk O2 saturation, % | 97 (97,98) | 97 (97,98) | 0.837 |
| Six-min walk distances, m | 570 (547,590) | 570 (558,585.5) | 0.837 |
| Post-walk O_2_ saturation, % | 99 (98,99) | 97 (97,98.5) | 0.091 |
| Post-walk systolic blood pressure | 119 (113,139) | 129 (122.5,144.5) | 0.351 |
| Post-walk diastolic blood presure | 83 (78,90) | 85 (83.5,87.5) | 0.837 |
| Post-walk heart rate | 85 (81,91) | 101 (94,108.5) | 0.071 |
| *HRCT* |  |  |  |
| Severe imaging findings of HRCT | 2(25%) | 1(14.3%) | 1.000 |

The quantitative data are shown as median data and IQR data in brackets.

The occurrence data are shown as no. (%). Values indicate no. of positive results/total no. of patients with available assay results.

Between- group comparisons of continuous variables in patients with low and high richness were tested by Kruskal- Wallis test.

Statistically significance with a p-value ≤0.05 was marked as bold.

A p-value ≤0.05 was denoted as statistically significant.

Pulmonary function tests were expressed as a percent of the predicted value.

MEF 25%, mean expiratory flow at 25%; MEF 50%, mean expiratory flow at 50%; MEF 75%, mean expiratory flow at 75%; DLCO, diffusing capacity of the lung for carbon monoxide; DLCO/VA, diffusing capacity divided by the alveolar volume; FEF25%–75%, forced expiratory flow at 25%–75%; FEV1, forced expiratory volume in the first 1 s of expiration; FVC, forced vital capacity; IVC, inspiratory vital capacity; MVV, maximal voluntary ventilation; PEF, peak expiratory flow; PFTs, pulmonary function tests; RV, residual volume; RV/TLC, residual volume divided by the total lung capacity; TLC, total lung capacity.
